# Meta-Analysis of Large-Scale Toxicogenomic Data Finds Neuronal Regeneration Related Protein and Cathepsin D to Be Novel Biomarkers of Drug-Induced Toxicity

**DOI:** 10.1371/journal.pone.0136698

**Published:** 2015-09-03

**Authors:** Hyosil Kim, Ju-Hwa Kim, So Youn Kim, Deokyeon Jo, Ho Jun Park, Jihyun Kim, Sungwon Jung, Hyun Seok Kim, KiYoung Lee

**Affiliations:** 1 Department of Biomedical Informatics, Ajou University School of Medicine, Suwon, Korea; 2 Severance Biomedical Science Institute, Yonsei University College of Medicine, Seoul, Korea; 3 Department of Genome Medicine and Science, School of Medicine, Gachon University, Incheon, Korea; 4 Brain Korea 21 PLUS Project for Medical Science, Yonsei University College of Medicine, Seoul, Korea; Queen's University Belfast, UNITED KINGDOM

## Abstract

Undesirable toxicity is one of the main reasons for withdrawing drugs from the market or eliminating them as candidates in clinical trials. Although numerous studies have attempted to identify biomarkers capable of predicting pharmacotoxicity, few have attempted to discover robust biomarkers that are coherent across various species and experimental settings. To identify such biomarkers, we conducted meta-analyses of massive gene expression profiles for 6,567 *in vivo* rat samples and 453 compounds. After applying rigorous feature reduction procedures, our analyses identified 18 genes to be related with toxicity upon comparisons of untreated versus treated and innocuous versus toxic specimens of kidney, liver and heart tissue. We then independently validated these genes in human cell lines. In doing so, we found several of these genes to be coherently regulated in both *in vivo* rat specimens and in human cell lines. Specifically, mRNA expression of neuronal regeneration-related protein was robustly down-regulated in both liver and kidney cells, while mRNA expression of cathepsin D was commonly up-regulated in liver cells after exposure to toxic concentrations of chemical compounds. Use of these novel toxicity biomarkers may enhance the efficiency of screening for safe lead compounds in early-phase drug development prior to animal testing.

## Introduction

In the early phases of drug development, efficient methods of assessing the safety of new drugs are needed. Current toxicity assessments typically require excessive animal sacrifice, large quantities of the drug compound, and long-term testing [1]. Generally involving the observation of drug responses in animals and the extrapolation thereof to humans, these assessment methods can be expensive, time consuming, and low in throughput [2]. Accordingly, demand for *in vitro* methods capable of predicting compound toxicity in humans is growing. Recently, molecular biomarker-based methods of predicting toxicity have gained traction for their potentially greater speed and accuracy compared to conventional methods [3].

In toxicogenomics, researchers seek to identify reliable molecular markers whose expression is tightly coupled to the development of specific target organ/systemic toxicity [3]. Historically, the rat has been the preferred model system for identifying organ-specific markers that respond to a wide variety of clinical compounds [4]. For example, in rats, 19 genetic biomarkers, including Kim1 (kidney injury molecule-1) and Spp1 (secreted phosphoprotein 1), and 35 genes, including Grik4 (glutamate receptor, ionotropic kainite 4) and Hspb7 (heat shock 27kDa protein family, member 7), have been identified as markers for kidney toxicity [5,6], while a 200-gene signature has been discovered for liver toxicity [7]. Alternatively, *in vitro* model systems utilizing human cell lines have also identified *EGR1* (early growth response 1), *ATF3* (activating transcription factor 3), *GDF15* (growth differentiation factor 15), and *FGF21* (fibroblast growth factor 21) to be biomarkers of drug toxicity, based on responses to 158 clinical compounds [8].

Nevertheless, although previous toxicogenomic studies have identified many candidate genes, their results are typically limited to specific compounds and the context in which they were derived (i.e., species and experimental setting) [9]. This raises major challenges in discerning the coherence between *in vitro* and *in vivo* settings, the appropriateness of the use of animal-derived markers in humans, and the robustness of a biomarker to different types of chemical perturbations. To address these challenges, we conducted a meta-analysis of publicly available toxico-transcriptomic datasets, followed by stepwise feature-selection procedures, to identify molecular biomarkers that respond robustly to a broad range of drugs in both *in vivo* rat specimens and *in vitro* human cell lines. Importantly, using computational and experimental cross-validation, we identified two novel toxicity molecular biomarkers, neuronal regeneration related protein (*NREP*) and cathepsin D (*CTSD*), as holding distinct prediction capabilities. As these marker proteins exhibit the ability to forecast *in vivo* toxicity and are easily detected in human cell line models, we believe that they can be important additions to existing toxicity assessment, with the potential to filter or prioritize drug candidates in the early stages of drug development.

## Materials and Methods

### Workflow

**Fig 1:**
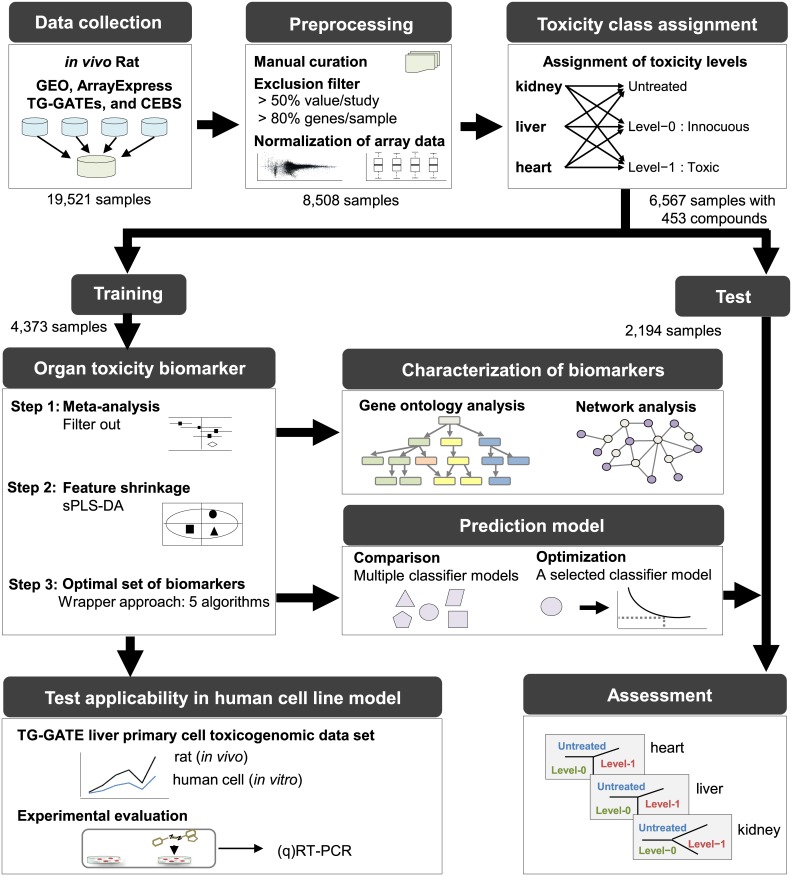
Overview of toxicity biomarker discovery. First, we collected toxicogenomic meta-data from public resources, preprocessed gene expression array data, and assigned toxicity classes. Second, we attempted to identify differentially expressed genes (DEGs) through meta-analysis and subsequent multistage feature reductions. DEGs were subjected to systems analysis of biological pathways and networks, and an optimized set of biomarkers was used to generate and validate a prediction model. The final step involved computationally and experimentally testing the applicability of the discovered biomarkers in human cells. GEO, Gene Expression Omnibus at the National Center for Biotechnology Information; ArrayExpress, ArrayExpress at the European Bioinformatics Institute; TG-GATEs, Toxicogenomics Project-Genomics Assisted Toxicity Evaluation System of the National Institute of Health Sciences of Japan; CEBS, Chemical Effects in Biological Systems at the National Institute of Environmental Health Sciences; sPLS-DA, sparse partial least squares discriminant analysis.

Our data mining and experimental workflow consisted of three main stages: 1) data preparation, 2) computational identification of biomarker candidates and generation/validation of the prediction model, and 3) human cell line-based evaluation of biomarker candidates. The workflow is presented in Fig 1 and is explained in detail below.

### Data collection and preprocessing

We initially performed a keyword-based search and downloaded toxicity-related gene expression profiles with associated pathology information from *in vivo* rat studies stored in multiple repositories and databases. The following keywords were utilized: organ toxicity, kidney toxicity, nephrotoxicity, liver toxicity, hepatotoxicity, heart toxicity, cardiac toxicity, brain toxicity, neurotoxicity, blood toxicity, hemotoxicity, lung toxicity, respiratory system toxicity, skin toxicity, dermotoxicity, phototoxicity, immune system toxicity, immunotoxicity, ocular and visual system toxicity, phototoxicity, endocrine system toxicity, and pituitary toxicity. We collected gene expression profiles (n = 19,521) from the following resources: i) Gene Expression Omnibus (GEO) at the National Center for Biotechnology Information [10], ii) ArrayExpress at the European Bioinformatics Institute [11], iii) Chemical Effects in Biological Systems (CEBS) at the National Institute of Environmental Health Sciences [12], and iv) the Toxicogenomics Project-Genomics Assisted Toxicity Evaluation System (TG-GATEs) of the National Institute of Health Sciences of Japan [13]. Various microarray platforms from Affymetrix Inc. (Santa Clara, CA), Agilent Technologies Inc. (Santa Rosa CA), Illumina Inc. (San Diego, CA), and GE Healthcare/Amersham Biosciences (Tempe, AZ) were used for these studies. Affymetrix CEL data format files were quantile normalized using the Robust Multiarray Averaging (RMA) method [14]. Background-subtracted median intensity values for Agilent and pre-processed values for Illumina and GE Healthcare/Amersham data were quantile normalized. After additionally downloading related data, including experimental conditions and pathology results, we conducted in-depth manual curation of the experimental conditions, based on the ToxRefDB format [15], and mapped histopathological descriptions of specific organs to standardized specific pathology codes (S1 Table). For toxicity class assignment, we used the pathology scores assigned by the individual studies: namely, 0 (within normal limits), 1 (minimal), 2 (mild), 3 (moderate), or 4 (severe), based on the pathology code of the corresponding organ and according to previous guidelines [16]. We focused on kidney, liver, and heart tissue, which are the primary organs used in research on drug-induced organ injury. We excluded genes with > 50% missing values in each study. We also excluded samples with > 80% missing genes [17]. After filtering out samples and genes with missing values, inter-array gene expression values were quantile normalized for 8,508 sample arrays (1,335 kidney, 6,491 liver, and 682 heart specimens) to make the entire dataset coherent in distribution. No further correction of nonsystematic variation from between-batch effects was implemented, since the datasets for our study were originally generated by several different groups, who used a wide variety of chemical perturbations, and thus, are highly confounded with numerous sources of potential batch effect.

### Toxicity class assignment

The toxicity class of each sample was assigned using the average of severity scores for the mapped histopathological codes. For this, we used the partial least squares discriminant analysis (PLS-DA) approach [18] implemented in the mixOmics R package [19] to determine the optimal threshold value of the average severity score that minimizes the classification error rate in a 10-fold cross-validation (S1 Fig). PLS-DA is one of the most popular classification techniques for generating a multivariate model that maximizes the discrimination between pre-defined sample groups [20]; however, the approach is prone to overfitting that requires rigorous internal validation by cross-validation and/or externally with an independent dataset. In the present study, the PLS-DA classification model was built for the top 300 DEGs from the meta-analysis for each of the discretized threshold values. To calculate the error rate of the PLS-DA classification model, a three-dimensional projection space was considered (S1 Fig). Further details on the meta-analysis are listed below. Using the optimal threshold of 0.5, we assigned 6,567 of the 8,508 samples with histopathological information on 453 human drugs to one of three toxicity classes: i) untreated, normal samples showing a lower average toxicity score than the threshold; ii) level-0, drug treated with no or minor histopathological phenotype with an average toxicity score below the threshold; and iii) level-1, drug treated with major toxicity phenotype and an average toxicity score above the threshold. Of the 6,567 samples, 4,373 were used for discovering biomarkers and generating a prediction model, and 2,194 were used for validation (S2 Table and S3 Table). Dosage and treatment schedule were not stratified to reduce the complexity of the analysis.

### Identification of DEGs by meta-analysis

For 4,373 training samples, we identified DEGs (i.e., candidate molecular biomarkers) using the random-effect meta-analysis method Hedges’ g, applying the standardized mean difference as an effect-size index in a stepwise manner [21]. The random-effect model assumes that all studies are heterogeneous; therefore, in assigning weights of studies, it simultaneously considers intra-study and inter-study variance. This characteristic of the random-effect model helps to reduce bias between studies, including batch effects, during analysis. Subsequent meta-analysis (MA) comparisons were used to identify DEGs for five target classes: (i) MA1, untreated versus treated to identify pan-organ treatment-specific DEGs; (ii) MA2, kidney versus liver versus heart specimens among drug-treated samples to identify organ-specific DEGs; and (iii) MA3, level-0 versus level-1 kidney specimens; (iv) MA4, level-0 versus level-1 liver specimens; and (v) MA5, level-0 versus level-1 heart specimens to identify organ-specific DEGs in accordance with toxicity responses. DEGs were identified in each comparison using a *p*-value cut-off of 0.0005. This *p*-value threshold was selected, because 44 known toxicity markers (S4 Table) [5, 9, 22–25] were most significantly enriched under this threshold and, at the same time, it provided a sufficiently large enough number of resultant DEGs for us to perform downstream analysis.

### Feature reduction by sparse PLS-DA and wrappers

Sparse PLS-DA (sPLS-DA) was performed to select the most discriminative genes among DEGs obtained by the MA comparisons for the training dataset. sPLS-DA achieved variable selection and classification in one procedure by iterating the following steps over discrete tuning parameters, such as sparsity and number of latent variables: i) generation of a multi-variate model using a given number of genes, ii) selection of pre-determined number of variables with the longest Euclidean distance, and iii) model-validation by 10 repetitions (10x) of 10-fold cross-validation. For classification, we considered the first three sparse PLS-DA (sPLS-DA) dimensions because it outperformed classifiers with one or two dimensions. When more than one gene shared distance rank, all were selected. sPLS-DA was conducted using the mixOmics R package [19].

A wrapper method was applied for further feature reduction using additional classification methods other than PLS-DA. A wrapper approach is powerful in identifying optimal variable subsets when the number of variables is relatively small [26]. Again, sPLS-DA was used to generate gene subsets for each of the five MA comparisons, followed by five different classifiers (linear discriminant analysis [LDA], random forest [RF], K-nearest neighbor [KNN], probabilistic K-nearest neighbors [PKNN], and support vector machine [SVM] methods), to find conditions with the lowest median classification error in a 10-fold cross-validation using different wrappers for each comparison. The optimal number of genes was determined by taking the median value of the numbers of DEGs from the five classifiers that showed the lowest error for each comparison (S2 Fig). The same Euclidean distance based method employed for sPLS-DA was used to identify the optimal set of DEGs.

### Prediction model generation and performance assessment

Using the optimal set of DEGs selected above, we compared the performances of the five classification models (LDA, RF, KNN, PKNN, and SVM). The accuracies of 10x, 10-fold cross-validations were averaged. The best performing classifier model with the lowest error rate was selected and further optimized with regard to the number and size of the decision tree. We assessed the performance of the generated model in 2,194 independent test samples and compared it with the performance of a model built with the 44 known genomic biomarkers.

### Gene Ontology and protein-protein interaction network analysis

Gene ontology (GO) analysis was performed with DEGs from the MA3 and MA4 comparisons against biological process terms using the Database for Annotation, Visualization and Integrated Discovery (DAVID) [27] to identify enriched biological functions in response to drug-induced toxicity in kidney and liver tissue. DEGs from MA5 (heart toxicity) were not considered since the number of DEGs from this comparison was not sufficiently large enough for GO analysis. Significantly enriched terms were identified using a *p*-value cut-off of 0.05. For network analysis, we used the Cytoscape plugin “Molecular Complex Detection” (MCODE) to identify protein-protein interaction (PPI) subnetworks [28]. The results were visualized using Cytoscape [29]. Rat PPIs from the following 13 sources were combined: BIND [30], BIND_t [30], BioGRID [31], CORUM [32], DIP [33], HPRD [34], IntAct [35], MINT [36], MPPI [37], OPHID [38], InnateDB [39], MatrixDB [40], and mentha [41]. Using the ortholog mapping relationships reported in the NCBI HomoloGene database [42], we combined the most recent human PPIs [43] with the rat PPIs. A total of 169,723 interactions involving 13,768 proteins were included in the network model.

### Analysis of a toxicogenomic dataset for human primary hepatocytes

A large-scale human cell-based pharmacotoxicity assay dataset is publically available for the liver. We obtained data from the TG-GATEs database [13], which contains a large-scale gene expression profile (n = 2,004 samples) and associated cell viability data (determined based on DNA content) for human primary hepatocyte cells treated with 158 chemical compounds. Information in this dataset was considered to be relevant to our biomarker candidates discovered in comparisons MA1 and MA4. Before the analysis, we first filtered out untreated samples with low DNA content (< 80%), which may represent contamination or some unknown environmental stress, and removed treated samples with > 100% DNA content to limit our search space to toxicity, not to hyper-proliferation. Application of these filters yielded a total of 572 untreated and 838 treated samples. In these samples, we examined the differential expressions of the five MA1 DEGs (*NREP*, *ATRN*, *TBXA2R*, *KIFC1*, and *EPHX1*) obtained in our study after all feature reductions (Table 1). Second, using varying thresholds of 76–98% DNA content, we assigned toxicity classes of level-0 (> = threshold) or level-1 (< threshold) to the treated samples, and further analyzed the differential expressions of the three MA4 DEGs (*CTSD*, *TPM4* and *RPL35A*) for liver toxicity obtained after feature reductions (Table 1). For the analysis, raw expression data (.cel format) were RMA normalized with the Affy R package.

**Table 1:** Number of selected genes after meta-analysis, sPLS-DA, and wrapper approaches for all five meta-analysis comparisons.

Comparison	Meta-analysis	sPLS-DA	Wrappers
Untreated *vs*. treated (MA1)	108	7	5
Organ *vs*. organ comparison (MA2)	3667	3	3
Level−0 *vs*. level−1 Kidney (MA3)	303	24	7
Level−0 *vs*. level−1 Liver (MA4)	661	3	3
Level−0 *vs*. level−1 Heart (MA5)	12	3	3
Total	4,023	40	21

### Cell lines and drug assays

HepG2 (human liver carcinoma) and HEK293 (human embryonic kidney) cell lines were purchased from American Type Tissue Collection (Rockville, MD, USA) and cultured in Dulbecco’s modified Eagle’s medium (DMEM) with 10% fetal bovine serum (FBS) under a humidified atmosphere with 5% CO_2_ at 37°C. HepG2 cells at 70–80% confluence were incubated for 48 h with 0–20 mM acetaminophen; HEK293 cells at 70–80% confluence were incubated for 72 h with 0–40 μM cisplatin. For these experiments, each of the two compounds was dissolved in both dimethyl sulfoxide (DMSO) and growth medium (DMEM with 10% FBS) before being added to cells. Cells incubated with 1% DMSO or growth medium served as controls. The effect of exposure to the respective compounds on cell viability was determined using MTS assay (Promega Corp., Madison, WI, USA), which estimates titers of metabolically active cells. Levels of toxicity for each cell line was determined based on MTS assay results: level-0, viability ≥ 60%, or level-1, viability < 60%. On the basis of preliminary MTS assay results, HepG2 cells were treated with 1 mM (level-0) and 10 mM (level-1 for DMSO) or 20 mM (level-1 for DMEM) acetaminophen for 48 h, and HEK293 cells were treated with 2 μM (level-0) or 20 μM (level-1) cisplatin for 72 h.

### Semi-quantitative RT-PCR and qRT-PCR

Total RNA was extracted using QIAzol lysis reagent (Qiagen, Hilden, Germany), according to the manufacturer’s instructions. Aliquots of total RNA (1 μg) were used to synthesize first-strand cDNA with Superscript reverse transcriptase (Invitrogen, Carlsbad, CA, USA) for PCR amplification. Semi-quantitative RT-PCR was then performed under the following conditions: 40 cycles of denaturation at 95°C for 15s, annealing at 60°C for 30 s, and extension at 72°C for 10 s, followed by a terminal extension at 72°C for 10 min. A house keeping gene, *GAPDH*, served as an internal control. Quantitative RT-PCR (qRT-PCR) was performed using the 7500 Real-Time PCR system (Applied Biosystems, Foster City, CA, USA) with Power SYBR Master Mix (Applied Biosystems). The thermocycling conditions used for the PCR experiments were 40 cycles of denaturation at 95°C for 15 s and extension at 60°C for 1 min. The following primer sequences for PCR were used: *NREP*, 5’-catgcactgcacttcttcgt-3’ and 5’-catgcactgcacttcttcgt-3’; *CTSD*, 5’-catgcactgcacttcttcgt-3’ and 5’-catgcactgcacttcttcgt-3’; *TPM4*, 5’-ttgaggaggagttggacagg-3’ and 5’-gctgcatctcctgaatctcc-3’; *TRPM4*, 5’-ccactgtcaggaccaccttt-3’ and 5’-ccccagtgtgaggaatctgt-3’; and *GAPDH*: 5’-gagtcaacggatttggtcgt-3’ and 5’-gacaagcttcccgttctcag-3’.

## Results

### Characterization of toxicogenomic meta-data

**Fig 2:**
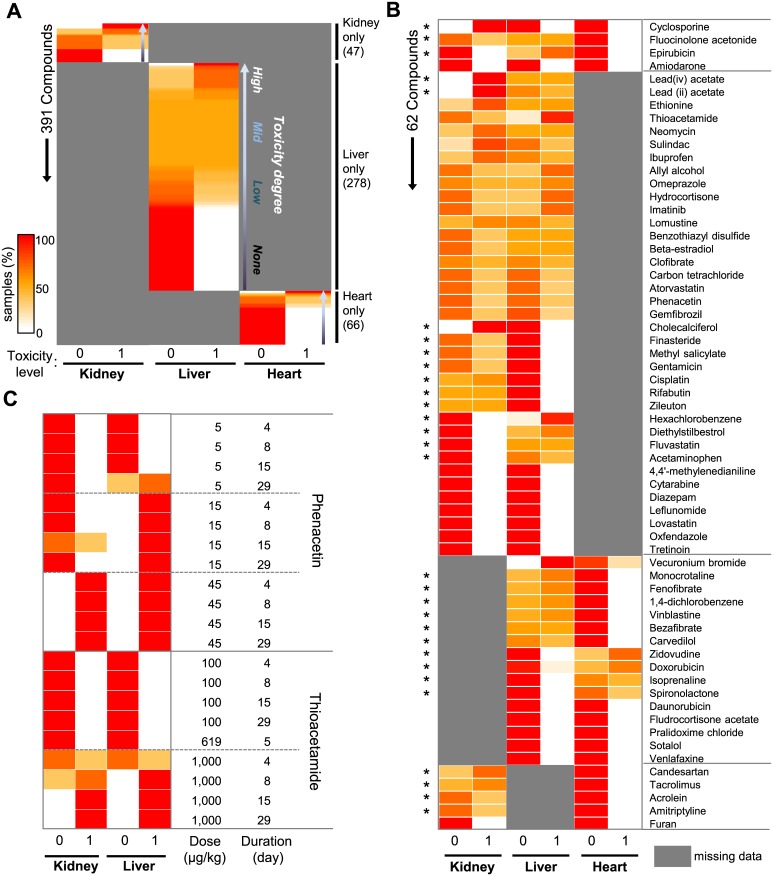
Characterization of pharmacogenomics meta-data. (A) Distribution of toxicity levels for 391 compounds from single-organ studies. Compounds were rank-ordered by relative toxicity level. (B) Distribution of toxicity levels for 62 compounds from multi-organ studies. Asterisks indicate compounds showing organ-specific toxicity. (C) Distribution of toxicity levels for two selected drugs at different doses and treatment durations. The same color scale is used in all panels. Missing information is shown in grey. For each row, the sum of samples with level-0 and level-1 toxicity per each organ is 100%. See S5 Table for the exact values used to generate this figure.

We characterized the 6,567 meta-data samples in terms of tested organs, drug identity, and treatment conditions (dose and duration) in relation to toxicity levels. Toxicity to 453 drugs was evaluated in kidney (n = 47), liver (n = 278), heart (n = 66), or multiple organ (n = 62) specimens (Fig 2A and 2B). Varying degrees of toxicity were observed for drugs tested in a single organ (Fig 2A and S5 Table). Drugs tested in multiple organs exhibited organ-selective toxicity (Fig 2B): for example, well-known nephrotoxins, such as cisplatin and gentamicin, were toxic to the kidney but not to the liver. Conversely, the well-known liver-damaging drugs acetaminophen and fluvastatin showed selective hepatotoxicity but not nephrotoxicity in our dataset (Fig 2B). As expected, dose-dependent and treatment time-dependent toxicity was also observed in our dataset (Fig 2C). Therein, phenacetin was toxic at 15 μg/kg and 45 μg/kg in the liver and kidney, respectively; meanwhile, thioacetamide induced kidney and liver toxicity in all samples at 1,000 μg/kg after 8–15 days of treatment (Fig 2C).

### Discovery of pharmacotoxicity biomarkers

**Fig 3:**
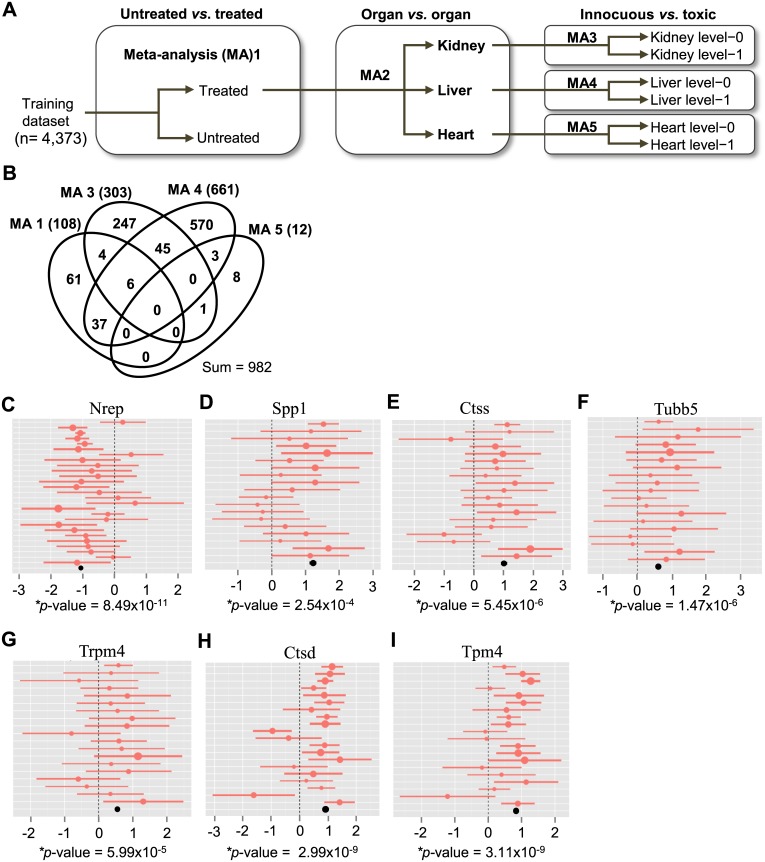
Meta-analysis identifies candidate biomarkers of organ toxicity. (A) Schematic flow chart of the meta-analysis. Untreated, untreated samples (pathology score < 0.5); level-0, innocuous treatment; level-1, toxic treatment. (B) Venn diagram for the overlap of DEGs identified from the four drug-related meta-analysis comparisons in (A). Numbers indicate gene counts. (C-I) Forest plots display the study-specific meta-analysis effect-sizes and 95% confidence intervals for the studies included in the training dataset. Plots for the seven DEGs from the MA1, MA3, and MA4 datasets with the greatest absolute average effect-size (> 0.55; See S8 Table) are shown. Plots for the remaining 11 DEGs are shown in S3 Fig. Nrep in untreated versus treated specimens; Spp1 (secreted phosphoprotein 1), Ctss (cathepsin S), Tubb5 (tubulin β5), and Trpm4 (transient receptor potential cation channel, subfamily M, member 4) in level-0 versus level-1 kidney specimens; and Ctsd (cathepsin D) and Tpm4 (tropomyosin 4) in level-0 versus level-1 liver specimens. The sizes of the circles are proportional to the fold-change (log2 ratio). The summarized effect-size (mean fold-change) of all enrolled studies is shown as a black circle at the bottom of the plot. *p*-value, Z-test for the overall effect of the summarized meta-analysis results for each gene.

Using a multi-step method, we attempted to identify robust molecular biomarkers of toxicity to 453 drugs among 4,373 meta-data samples. With these biomarkers, we then built a prediction model that was subsequently tested in an independent dataset of 2,194 samples (Fig 3A and S3 Table). First, we applied Hedges’ g statistic to identify DEGs across multiple orthogonal datasets that compared innocuous versus toxic drug treatments separately for kidney (MA3), liver (MA4), and heart (MA5) tissue. Two additional comparisons were conducted to detect primary responders to broad chemical perturbations in treated versus un-treated specimens for multi-organ datasets (MA1) and to distinguish organ-specific responses to drug treatment for liver, kidney, and heart specimens (MA2). DEGs discovered in MA1 and MA2 were considered along with DEGs from MA3-5 during feature selection so that the results from organ-specific comparisons of toxic versus non-toxic treatment in MA3-5 would be supported by those from comparison of pan-organ and organ-specific responses to drug treatment in MA1 and MA2, respectively.

Excluding the MA2 comparison, a total of 982 DEGs were found to be relevant to responses to drug treatment (Fig 3B). Intriguingly, about 30% of the DEGs from MA3, MA4, and MA5 were co-detected in more than one organ, suggesting their use in detecting toxicity in multiple organs. However, none of these DEGs was detected in all three organs, likely reflecting a lower detection power in the heart than in the kidney and liver. Some of the organ-selective DEGs identified here correspond to previously reported toxicity markers (S6 Table). For example, DEGs noted in MA3, such as kidney injury molecule-1 (Kim1), ceruloplasmin (Cp), clusterin (Clu), and secreted phosphoprotein 1 (Spp1), are known kidney toxicity markers [24]. DEGs in MA4 included known liver toxicity markers, such as heme oxygenase (decycling) 1 (Hmox1), cathepsin L1 (Ctsl) [9], receptor-interacting serine-threonine kinase 3 (Ripk3), solute carrier family 7 (cationic amino acid transporter, y+ system), member 1 (Slc7a1), and chemokine (C-C motif) ligand 2 (Ccl2) [23].

To identify an efficient, succinct, and robust set of markers with sufficient resolution power to discriminate target classes, we performed feature reduction using the 4,023 DEGs obtained therein. To this end, we applied sPLS-DA, a multivariate exploratory approach that is a computationally efficient one-stage variable-selection and classification method [19], to select the smallest number of features from each comparison that would minimize the average misclassification error rate using a 10-fold cross-validation. A total of 40 genes were selected from the five comparisons (S7 Table). We further used five wrapper methods, LDA, RF, KNN, PKNN, and SVM, in an attempt to further reduce features, especially for comparison MA1 and 3 (S2 Fig). Application of these methods resulted in the selection of 21 genes from the five comparisons (Table 1). This rigorous feature reduction was necessary to narrow down our discoveries to the most discriminative and smallest set of biomarkers for greater utility in multiplex gene expression assay platforms, where the number of biomarkers (probes) is often limited technically by the number of resolvable detection channels and economically by the cost per probe.

Fig 3C–3I) highlights seven of 18 genes with the highest fold-change values from the four drug-relevant comparisons. Of these, the top-ranked Spp1 and third-ranked Ctss are well-known kidney pharmacotoxicity markers [5]. The other five genes Nrep, Trpm4, Tubb5, Ctsd, and Tpm4 are all novel pharmacotoxicity biomarker candidates.

### Evaluation of the toxicity prediction model with a test dataset

We compared the performance of five different classification models built using the training samples and selected the best classifier to build a toxicity prediction model for the kidneys, liver, and heart. We applied LDA, RF, KNN, PKNN, and SVM as classification algorithms and assessed the prediction accuracy thereof via 10-fold cross-validation as a performance measure (Table 2). Among these methods, RF with 10-fold cross-validation achieved the best performance (82% correct classification); thus, it was selected as the final classifier. Next, we evaluated the performance of the 21-gene RF prediction model in 2,194 independent test samples in comparison to a model with an identical degree of complexity built using previously known organ-specific toxicity markers, which we compiled from various studies [5, 9, 22–25]. As shown in Table 2, the accuracy of our 21-gene RF prediction model was slightly higher (62%) than the model built with previously known biomarkers (60.5%).

**Table 2:** Performance of prediction models using the 21 identified differentially expressed genes.

	Classifier model	Feature	Accuracy
Training set	LDA	21 DEGs	74.7
	RF	21 DEGs	82
	KNN	21 DEGs	76.9
	PKNN	21 DEGs	73
	SVM	21 DEGs	77.4
Test set	RF	21 DEGs	62
	RF	21 genes selected among known biomarkers^#^	60.5*

^#^Comparison of the classification performances of RF models in independent test samples for our 21-gene model and a model of the same size comprising sPLS-DA-selected known genes (n = 44; S4 Table).

*Average of 1,000 iterations of the model building and validation procedures.

LDA: linear discriminant analysis, RF: random forest, KNN: K-nearest neighbor, PKNN: probabilistic K-nearest neighbors, SVM: support vector machine, DEG: differentially expressed genes, sPLS-DA: sparse partial least squares discriminant analysis

### Pathway and network level functional characterization of the differentially expressed genes

**Fig 4:**
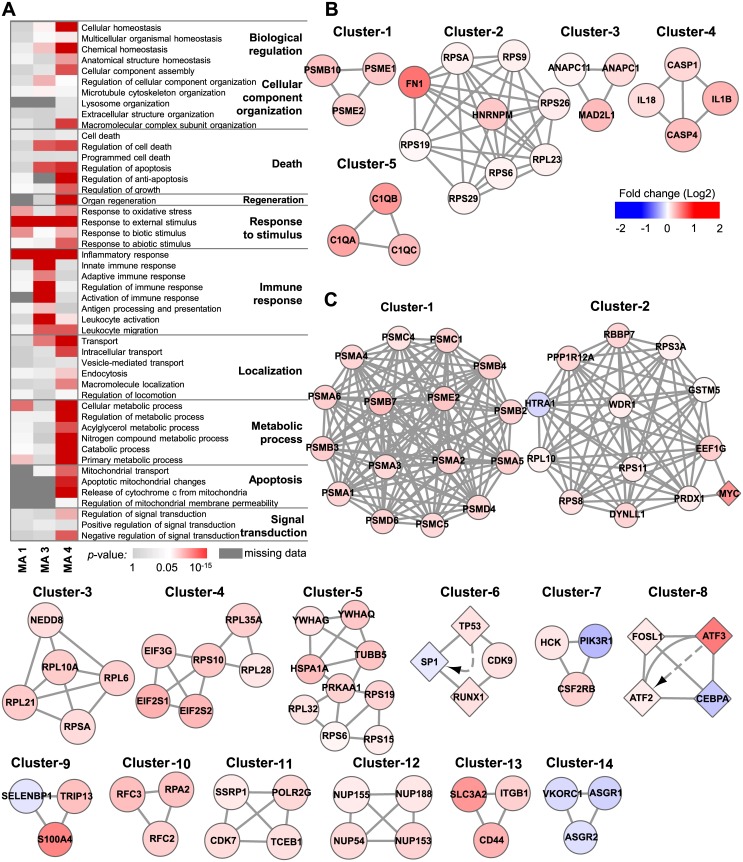
Functional analysis of DEGs. (A) Enriched GO terms associated with DEGs from three meta-analysis comparisons. DEGs from MA5 were excluded from the analysis owing to insufficient dataset size. *p*-value: modified Fisher’s exact test implemented in the Database for Annotation, Visualization and Integrated Discovery (DAVID). (B, C) Highly interconnected subnetworks present within the individual sets of DEGs from MA3 and MA4. A circular node indicates proteins, a diamond node indicates proteins/genes, and solid lines and dashed arrows respectively indicate physical and genetic interactions reported in our input databases (see Methods for details). Node color indicates the median expression fold-change of the training dataset (level-1/level-0).

To characterize underlying biological responses to chemical stress, we separately investigated enriched biological processes associated with DEGs discovered in the MA1, MA3, and MA4 comparisons by GO analysis. Due to a lack of available DEGs, MA5 (level-0 versus level-1 heart specimens) was excluded from the analysis. GO terms related to cell death, stress response, immune response, metabolic process, and signal transduction were identified (Fig 4A). Specifically, in the comparison of untreated versus treated specimens (MA1), the GO terms with the most significant *p*-values were “response to external stimulus” (*p* = 2.56×10^−5^), “inflammatory response” (*p* = 3.24×10^−5^), and “cellular metabolic process” (*p* = 5.57×10^−3^), whereas terms related to cell death or apoptosis were not significant, suggesting that this comparison detected gene expression signals relevant to the early responses to chemical stress prior to the development of pathophysiological responses. In contrast, DEGs associated with both kidney and liver toxicity responses were enriched for cell death and mitochondrial apoptosis, as well as “response to external stimulus” and “inflammatory response.” Interestingly, GO analyses also yielded liver-specific terms, such as “organ regeneration” (*p* = 1.05×10^−03^) and “cellular metabolic process” (*p* = 3.35×10^−15^). The liver is the only internal human organ capable of regeneration upon tissue loss or after acute toxic injury [44]. Moreover, one of the liver’s most important roles is to metabolize various xenobiotics. Accordingly, our DEGs well represent the biology of organ-specific responses to toxicity.

To further identify protein complexes within DEGs noted in MA3 and MA4 comparisons, we applied the cluster finding algorithm MCODE to the PPI network formed by the 303 genes from MA3 and the 661 genes from MA4 that corresponded with the DEGs. MCODE identified five clusters for MA3 and 14 clusters for MA4 (Fig 4B and 4C). Notably, proteasomes and ribosomes were identified for both MA3 and MA4. Cluster-1 from MA3 and cluster-1 from MA4 are different subsets of the giant proteasome complex. Proteasome mediated degradation of damaged proteins is an important defense mechanism against xenobiotic-driven reactive oxygen species-mediated stress [45–47]. Meanwhile, cluster-2 from MA3 and cluster-2, -3, -4, and -5 from MA4 are part of the ribosomal complex. Up-regulation of ribosomal machinery is also a known cellular defense mechanism against various genotoxic compounds that restores homeostasis by activating translation [48].

Different PPI components from DNA damage response-related processes were also discovered for both comparisons, namely, cluster-3 from MA3 and cluster-6, -7, -8, -9, -10, and -11 from MA4. Consistent with the GO analyses, MCODE identified protein complexes involved in inflammatory responses, including caspase-1-induced activation of interleukins (IL)-1B and IL-18 (cluster-4 of MA3), elevated components of complement (cluster-5 of MA3), and β-colony-stimulating factor 2 receptor (CSF2RB) complex (cluster-7 of MA4). Notably, complement activation is associated with various organ injuries, including acetaminophen-induced liver injury [49], and CSF2RB is a high-affinity receptor for IL-3, IL-5, and colony-stimulating factor [50]. Intriguingly, we identified several liver-specific PPI subnetworks, including nucleoporin (cluster-12) and hepatic lectin (cluster-14 of MA4), the latter of which is down-regulated in the liver upon exposure to various drugs. As a transmembrane protein, hepatic lectin is a known target of liver-specific drug delivery that internalizes receptor-bound molecules and viruses through endocytosis [51]. Thus, its down-regulation may possibly reflect a defense mechanism against hepatotoxins.

### *NREP* and *CTSD*: novel biomarkers of toxicity in human cell lines

Recapitulation of our biomarker candidates in appropriate human cell models would potentially allow for their adoption into prediction of toxicity for drug candidates early in drug development. Accordingly, the following two-track approach was undertaken to identify toxicity markers applicable to human cell lines: 1) evaluating the performance of eight of the 21 candidate markers from MA1 (untreated versus treated) and MA4 (level-0 versus level-1 liver specimens) in TG-GATEs, a massive *in vitro* toxicogenomic dataset for human hepatocytes [13]; 2) experimentally testing expression changes for five of the 21 candidate markers from MA1, MA3, and MA4 that were selected based on pooled effect-size (> 0.55) after exposure to relevant pharmacological compounds in the human cell lines HEK293 and HepG2. Three of the 21 candidate genes from MA5 were not included because a human heart cell line is not commercially available (S8 Table).

**Fig 5:**
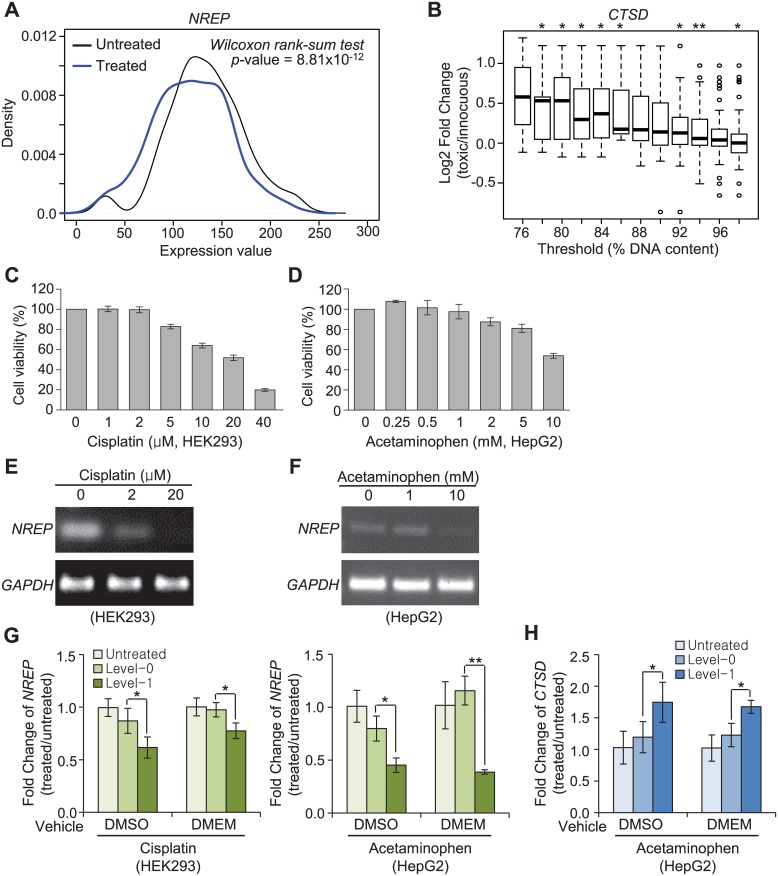
Computational and experimental validations identify NREP and CTSD as biomarkers of toxicity in human cell lines. (A) Density plots comparing expression levels of *NREP* between untreated and treated samples of liver primary hepatocytes reported in TG-GATEs. (B) Boxplots display fold-changes in *CTSD* (toxic/innocuous) at each of the given cell viability thresholds measured for the liver primary hepatocytes reported in TG-GATEs. * t-test *p*-value < 0.05, ** < 0.001. (C, D) Dose-responsive viability of HEK293 (C) and HepG2 (D) cells exposed to cisplatin (C) or acetaminophen (D). DMSO was used to dissolve the compounds. Cell viability was measured by MTS assay. Error bars represent ± standard deviation of triplicate experiments. See S4A and S4B Fig for the results with the same compounds dissolved in growth media. (E, F) *NREP* mRNA levels after exposure to the indicated concentrations of cisplatin for 72 h and acetaminophen for 48 h, respectively, determined by RT-PCR. (G-H) qRT-PCR assays for *NREP* (G) and *CTSD* (H). Y-axis indicates fold-changes in expression compared to chemically untreated samples (n = 5). Level-0 and level-1 drug concentrations for DMSO and DMEM were selected based on cell viability of > or < 60%, respectively, in C-D and S4A and S4B Fig. *p < 0.05, ** *p* < 0.001; Student’s t-test. Error bars represent ± standard deviation.

TG-GATEs comprises 1,410 transcriptome profiles and associated cell titers for human primary hepatocytes exposed to 119 pharmacological compounds. For this analysis, we first performed t-tests for differences in expression of the five early-toxicity marker candidates, *NREP*, *ATRN*, *TBXA2R*, *KIFC1*, and *EPHX1*, between untreated and treated samples. Of these, only *NREP*, which had the largest fold-changes in the meta-analysis (S8 Table), showed consistent and statistically significant depletion upon drug treatment (Fig 5A). Second, fold-changes in the expression of each of the three liver marker candidate genes, *CTSD*, *TPM4*, and *RPL35A*, were estimated using varying toxicity thresholds determined by relative cell titers (Fig 5B). Of these candidates, *CTSD* was found to be a statistically significant positive toxicity marker that showed an increasing fold-change with decreasing cell viability threshold (Fig 5B).

In parallel, we experimentally evaluated five novel toxicity biomarker candidates, *NREP*, *TUBB5*, *TRPM4*, *CTSD*, and *TPM4*, which showed the largest pooled effect-size (> 0.55) in RF analysis (S8 Table), in the human cell lines HEK293 and HepG2. In comparison to innocuous samples (level-0), *NREP*, a DEG identified from the comparison of untreated and treated specimens, was markedly down-regulated in both HEK293 (Fig 5C, 5E and 5G) and HepG2 cells (Fig 5D, 5F and 5G) treated with toxic concentrations (level-1) of known organ-selective toxins. Expression of *CTSD*, a DEG identified from the comparison of level-0 and level-1 liver specimens (MA4), was significantly elevated upon exposure to toxic concentrations of the liver toxin acetaminophen (Fig 5D and 5H). Notably, the observed expression changes in the two validated biomarker candidates were found to be robust against the type of vehicle used for drug preparation (DMSO or growth medium) (Fig 5G and 5H).

On the contrary, *TRPM4* and *TPM4*, DEGs discovered in comparison of level-0 and level-1 kidney specimens (MA3) and liver specimens (MA4), respectively, did not show consistent expression changes in relevant human cells treated with toxic concentrations of the drug compounds (S4E and S4F Fig). *TUBB5* showed no changes in gene expression in HEK293 cells (data not shown).

Importantly, *NREP* and *CTSD* were cross-validated by a two-track approach in human cells: computationally validated *in silico* via TG-GATEs and experimentally validated *in vitro* in the human cell lines HEK293 and HepG2. We found that *NREP* is a multi-organ biomarker for a wide variety of chemical perturbations and is down-regulated in response to drug-induced toxicity. We also found that *CTSD* is a liver-specific biomarker of toxicity that is concurrently induced with the onset of a pathological phenotype following various chemical perturbations.

## Discussion

Biomarkers of drug-induced toxicity enable cost effective pre-clinical evaluation of drug candidates in cell line models; however, their use is currently limited by a lack of markers robustly applicable to a wide variety of chemical compounds and predictive of *in vivo* pathological outcomes.

In the present study, we utilized massive toxicogenomic meta-datasets from various *in vivo* rat studies covering 453 pharmacological compounds at different doses and durations and accompanied by histopathological information. With these datasets, we performed meta-analysis, followed by multiple feature reduction procedures, to identify organ-specific biomarker candidates of drug-induced toxicity (Table 3). Subsequent *in silico* and *in vitro* analyses in human cell lines validated *NREP* and *CTSD* as strong biomarkers of drug-induced toxicity. The canonical functions of *NREP* include cell migration through activation of RalA in the initial wound matrix of proto-myofibroblasts and myofibroblasts [52] and wound healing in human and mouse cell lines [53, 54]. CTSD is a lysosomal aspartic endopeptidase known to mediate apoptosis in response to oxidative stress [55]. In the present study, *NREP* was down-regulated in response to general chemical stress and coupled to the onset of multi-organ toxicity. As well, *CTSD* was found to be up-regulated upon drug-induced toxicity in the liver.

**Table 3:** List of the 21 candidate biomarkers of drug-induced toxicity.

Comparison	GeneSymbol	Official Name from HGNC	Functional Categories
Untreated *vs*. treated (MA1)	Ephx1	epoxide hydrolase 1, microsomal (xenobiotic)	activation and detoxification of epoxides
	Atrn	attractin	cell survival
	Nrep	neuronal regeneration related protein	cell migration
	Kifc1	kinesin family member C1	proplatelet formation
	Tbxa2r	thromboxane A2 receptor	inflammation
Organ *vs*. organ comparison (MA2)	Azgp1	zinc-alpha-2-glycoprotein 1	cell proliferation, kidney injury
	Nrg1	neuregulin 1	development of multiple organ systems
	Hao2	hydroxyacid oxidase 2	peroxisomal enzyme, expressed predominantly in liver and kidney
Level−0 *vs*. level−1 Kidney (MA3)	Spp1	secreted phosphoprotein 1	inflammation, oxidative stress, fibrosis
	Ctss	cathepsin S	inflammation
	Tubb5	beta 5 tubulin	immune response
	Trpm4	transient receptor potential cation channel, subfamily M, member 4	T-cell activation
	Amigo2	adhesion molecule with Ig-like domain 2	apoptosis
	Il1rl2	interleukin 1 receptor-like 2	immune response
	Atp1b2	sodium/potassium-transporting ATPase subunit beta-2	ion homeostasis
Level−0 *vs*. level−1 Liver (MA4)	Tpm4	tropomyosin 4	repair and regeneration
	Ctsd	cathepsin D	immune response, apoptosis, oxidative stress
	Rpl35a	ribosomal protein L35A	cytotoxic damage
Level−0 *vs*. level−1 Heart (MA5)	Gpam	glycerol-3-phosphate acyltransferase, mitochondrial	proliferation
	Pcp4l1	purkinje cell protein 4 like 1	
	Rxrg	retinoid X receptor gamma	hematopoietic stem cell differentiation

HGNC: HUGO Gene Nomenclature Committee

Herein, the compilation of the large number of relevant toxicogenomic studies enabled us to perform meta-analyses capable of identifying robust prediction biomarkers that were otherwise undiscovered in the individual studies because of their limited statistical power. Nevertheless, combining different studies inevitably incorporates experimental biases. Potential sources of bias in toxicogenomic meta-analyses include experimental model selection, drug selection, organ selection, and pathological phenotype selection. In the present study, to tackle the bias in experimental model selection, both *in vivo* rat models and human cell line models were employed in the initial discovery and subsequent validation, respectively. Several previous studies have indeed shown that these types of pre-clinical models successfully predict toxicity in humans [56, 57]. Regarding the potential bias in drug selection, among 453 unique compounds compiled in our study, the three most-frequently tested drugs were acetaminophen (n = 191, 2.9%), diquat dibromide (n = 142, 2.2%) and 1,4-dichlorobenzene (n = 80, 1.2%), indicating that our study is not heavily biased in favor of only a few compounds. To avoid potential organ bias in combined analyses, we conducted separate analyses for three major organs: kidney (n = 1,140), liver (n = 4,813), and heart (n = 614). With regard to possible pathological phenotype bias, our compiled dataset was annotated with comprehensive organ-specific histopathology terms, as shown in S1 Table. The most frequently used terms were “infiltration, cellular” (n = 4,701, 71.6%) and “necrosis” (n = 3,599, 54.8%).

Additional challenges associated with heterogeneous sources of data involve addressing nonsystematic variation originating from between-batch effect, such as laboratory conditions, reagent lots, and personnel differences [58]. Although, several available algorithms are able to correct for batch effects, these would not be appropriate or efficient for use in studies such as ours, since our compiled datasets originated from studies of differing experimental conditions and of many different chemical stress conditions. Thus, the studies would be highly confounded with several potential sources of batch effect. The presence of nonsystematic variation may result in false positives and/or false negatives. Between these, false positives were the greatest concern in our study. To address this concern, we attempted to validate positive results experimentally in human cell lines. In doing so, we identified and validated two toxicity biomarkers that were highly robust against drug-induced toxicity.

In our study, organ-specific interactions were assessed only for toxicity levels (level-0 versus level-1) not for treatment status (untreated versus treated), as the focus of our study was to identify candidate biomarkers that could predict organ-specific toxicity rather than to outline organ-specific responses to chemical stress. Accordingly, we initially attempted to discover DEGs in MA1 without considering organ-specificity, since, when identified, these DEGs may serve as ubiquitous chemical stress markers to help interpret the results of organ-specific comparisons (MA3-5). *NREP*, which was identified from MA1, was both computationally and experimentally validated in our study as a multi-organ responder to chemical stress coupled to toxic phenotype.

Not all of the biomarkers identified *in vivo* were recapitulated in human cells. Only two of eight genes were reproduced in an orthogonal liver cell line toxicogenomic dataset, and four of five genes were experimentally validated by qRT-PCR in human cell lines. This limited coherence may originate from differences in species and between *in vivo* and *in vitro* experimental settings. One potential difference in the experimental settings that may have affected our results was the type of vehicle used for drug preparation. DMSO, a popular cryoprotectant for long-term cell storage, is widely used as a solvent and vehicle for many pharmaceutical compounds, especially in *in vitro* settings. Frequently, the effect of DMSO on drug responses and toxicity is ignored. Nevertheless, in our study, we experimentally confirmed that the two drug-induced toxicity biomarkers were not affected by the type of drug-solvent.

In this study, we identified and validated two novel gene expression biomarkers that were found to be predictive of drug-induced toxicity. If incorporated into an existing panel of biomarkers or further developed into an independent cell line-based molecular assay system, these biomarkers might enable guided design of lead compounds, obviating the need to test large numbers of drugs in animals in order to evaluate *in vivo* toxicity. This would improve the overall efficiency of drug-development processes.

## Supporting Information

S1 Fig Determination of the PLS-DA threshold of the average toxicity score for class assignment. (PDF)

S2 Fig Feature reduction by wrappers. (PDF)

S3 Fig Meta-analysis identifies toxicity biomarker candidates (second tier), related to Fig 3C–3I. (PDF)

S4 Fig Experimental validations identify NREP and CTSD as biomarkers of toxicity in human cell lines, related to Fig 5. (PDF)

S1 Table List of curated pathology terms for each organ according to the standardized ToxRefDB vocabulary. (PDF)

S2 Table List of samples for meta-analysis. (XLSX)

S3 Table Summary of the analyzed meta-data. (PDF)

S4 Table The 44 previously known biomarkers. (PDF)

S5 Table Distribution of compound selective toxicity levels in meta-data, related to Fig 2. (XLSX)

S6 Table List of DEGs identified in each meta-analysis. (XLSX)

S7 Table List of 40 genes selected from sPLS-DA. (PDF)

S8 Table Summary statistics for the 18 toxicity biomarker candidates. (PDF)
