# Correction: Meta-Analysis of Large-Scale Toxicogenomic Data Finds Neuronal Regeneration Related Protein and Cathepsin D to Be Novel Biomarkers of Drug-Induced Toxicity

**DOI:** 10.1371/journal.pone.0163403

**Published:** 2016-09-15

**Authors:** Hyosil Kim, Ju-Hwa Kim, So Youn Kim, Deokyeon Jo, Ho Jun Park, Jihyun Kim, Sungwon Jung, Hyun Seok Kim, KiYoung Lee

In the Funding section, the grant number from the funder KHIDI is listed incorrectly. The correct grant number is: HI14C1324.
