# Supplementary material for: Meta-Analysis of Large-Scale Toxicogenomic Data Finds Neuronal Regeneration Related Protein and Cathepsin D to Be Novel Biomarkers of Drug-Induced Toxicity
Source: PLoS One. 2015 Sep 3;10(9):e0136698. doi: 10.1371/journal.pone.0136698 (PMC4559398; doi:10.1371/journal.pone.0136698)
Supplement: S1 Fig — (PDF) [file pone.0136698.s001.pdf]

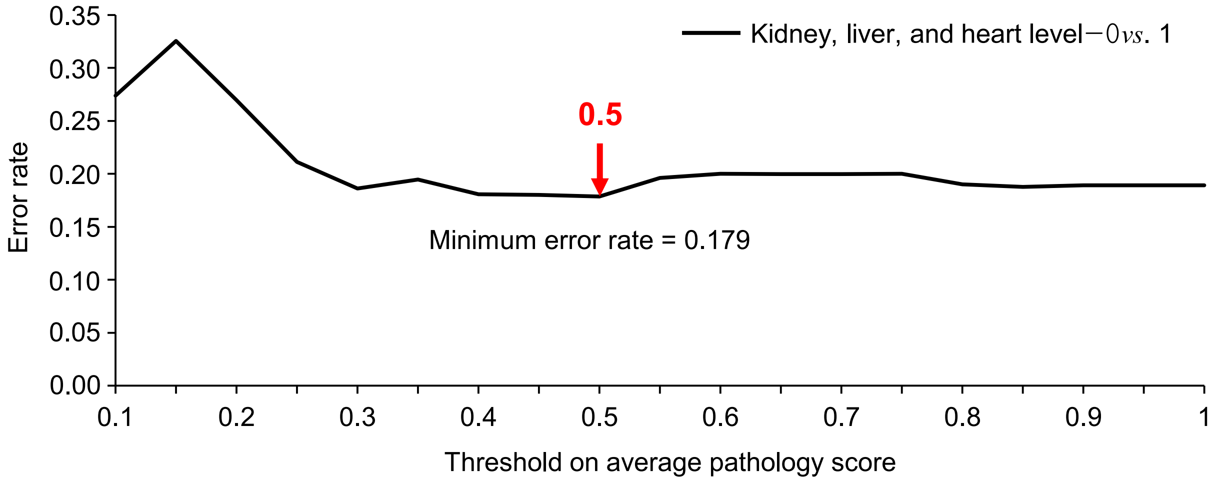

S1 Fig. Determination of the PLS-DA threshold of the average toxicity score for class assignment. The error rate was estimated by averaging 10 repetitions (10x) of the 10-fold cross-validation. The selected threshold for classifying toxicity levels is indicated by a red arrow.
