# Supplementary material for: Meta-Analysis of Large-Scale Toxicogenomic Data Finds Neuronal Regeneration Related Protein and Cathepsin D to Be Novel Biomarkers of Drug-Induced Toxicity
Source: PLoS One. 2015 Sep 3;10(9):e0136698. doi: 10.1371/journal.pone.0136698 (PMC4559398; doi:10.1371/journal.pone.0136698)
Supplement: S2 Fig — (PDF) [file pone.0136698.s002.pdf]

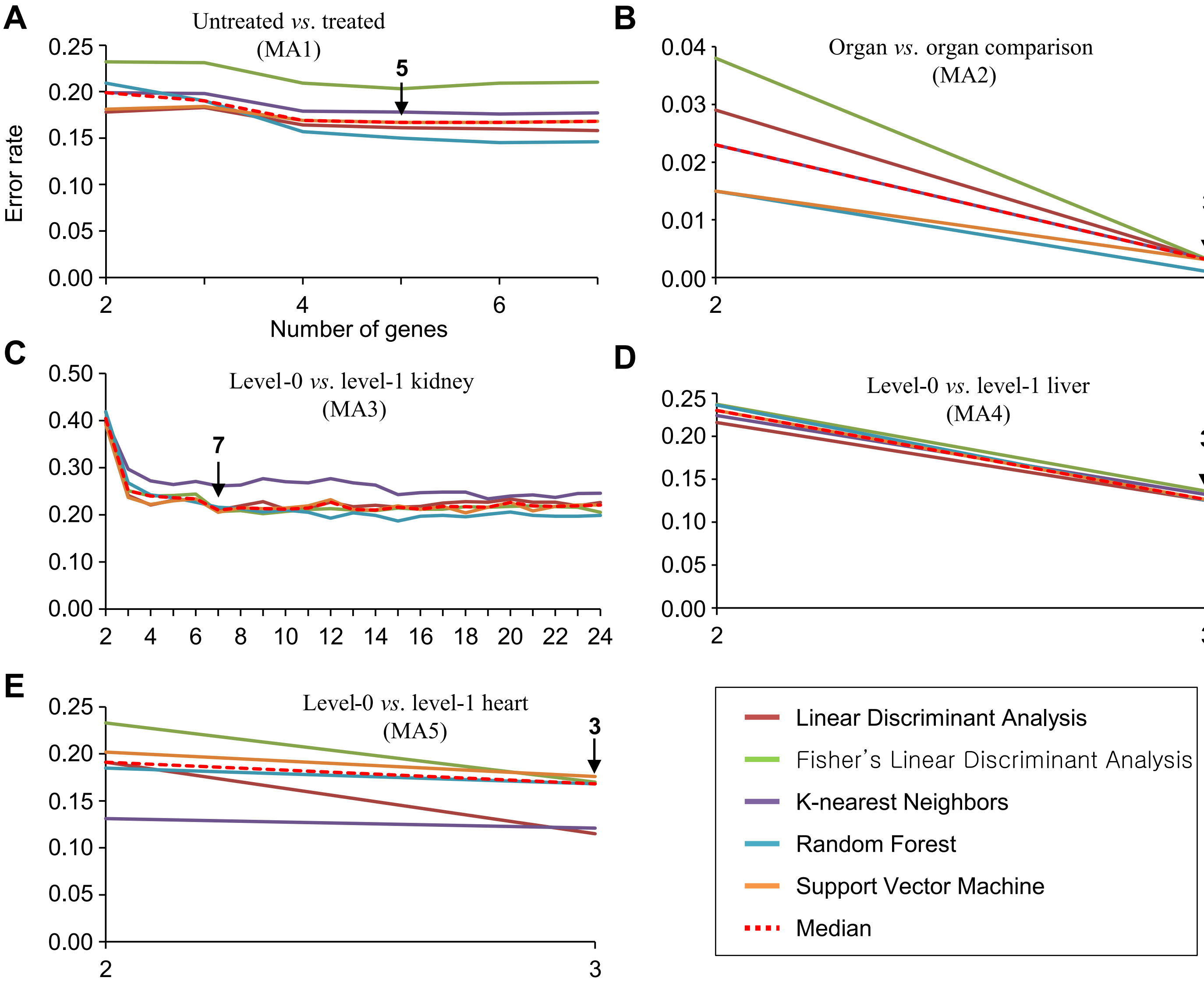

S2 Fig. Feature reduction by wrappers. Plots report the classification error rate for each of the five wrappers and their median error rate as a function of numbers of genes. The classification error rate is the average of five repetitions of a 10-fold cross-validation scheme measured for MA1 (A), MA2 (B), MA3 (C), MA4 (D), and MA5 (E). The optimal gene-number with the minimum-median error rate is indicated with an arrow.
