# Supplementary material for: Meta-Analysis of Large-Scale Toxicogenomic Data Finds Neuronal Regeneration Related Protein and Cathepsin D to Be Novel Biomarkers of Drug-Induced Toxicity
Source: PLoS One. 2015 Sep 3;10(9):e0136698. doi: 10.1371/journal.pone.0136698 (PMC4559398; doi:10.1371/journal.pone.0136698)
Supplement: S3 Fig — (PDF) [file pone.0136698.s003.pdf]

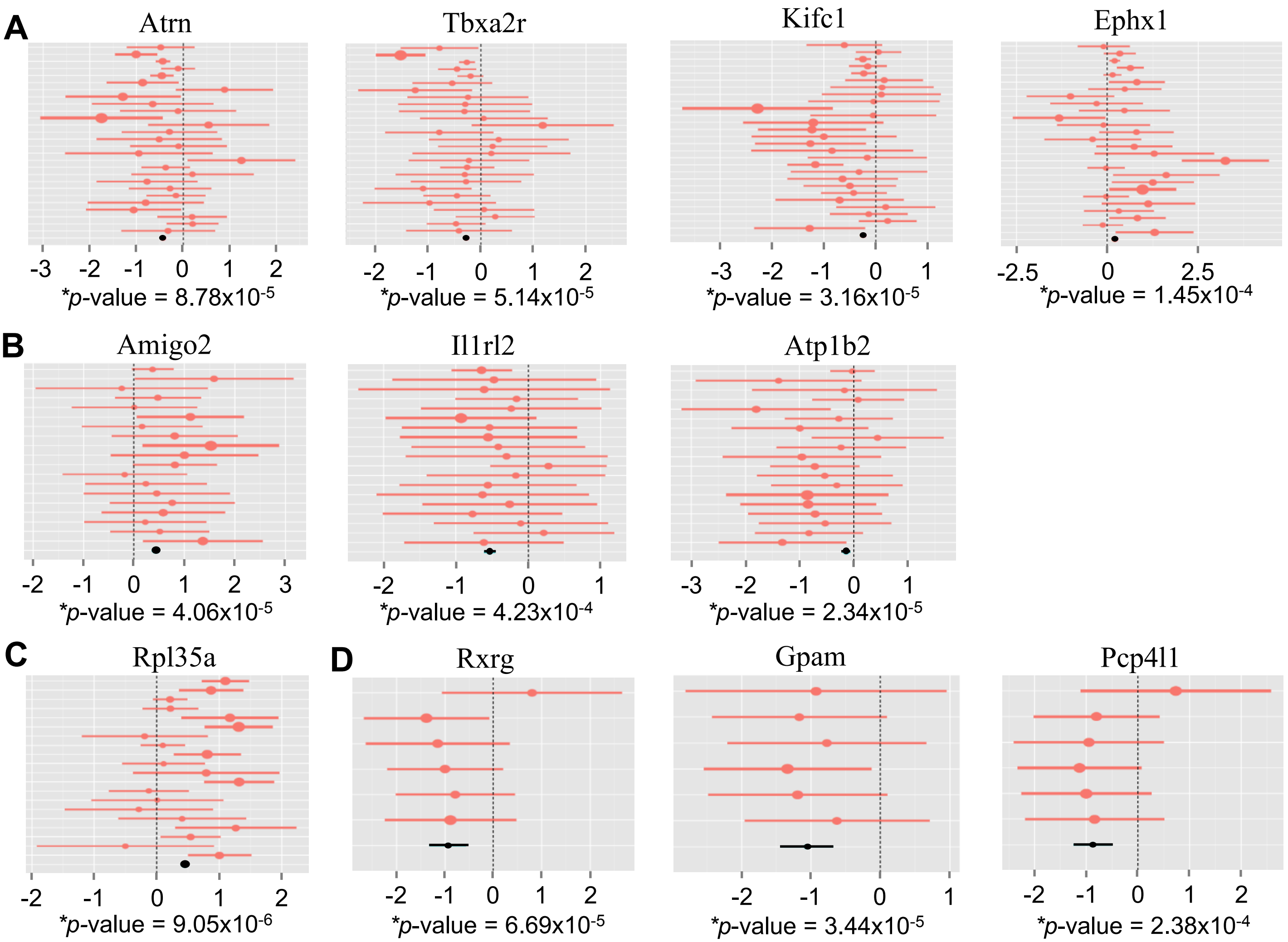

S3 Fig. Meta-analysis identifies toxicity biomarker candidates (second tier), related to Fig 3C-I. Eleven of the 18 markers that are not shown in Fig 3C-I are displayed as forest plots from each of the four comparisons: untreated vs. treated (A), level-0 *vs*. level 1 kidney (B), level-0 *vs*. level-1 liver (C), and level-0 *vs*. level-1 heart (D).
