# Supplementary material for: Meta-Analysis of Large-Scale Toxicogenomic Data Finds Neuronal Regeneration Related Protein and Cathepsin D to Be Novel Biomarkers of Drug-Induced Toxicity
Source: PLoS One. 2015 Sep 3;10(9):e0136698. doi: 10.1371/journal.pone.0136698 (PMC4559398; doi:10.1371/journal.pone.0136698)
Supplement: S4 Fig — (PDF) [file pone.0136698.s004.pdf]

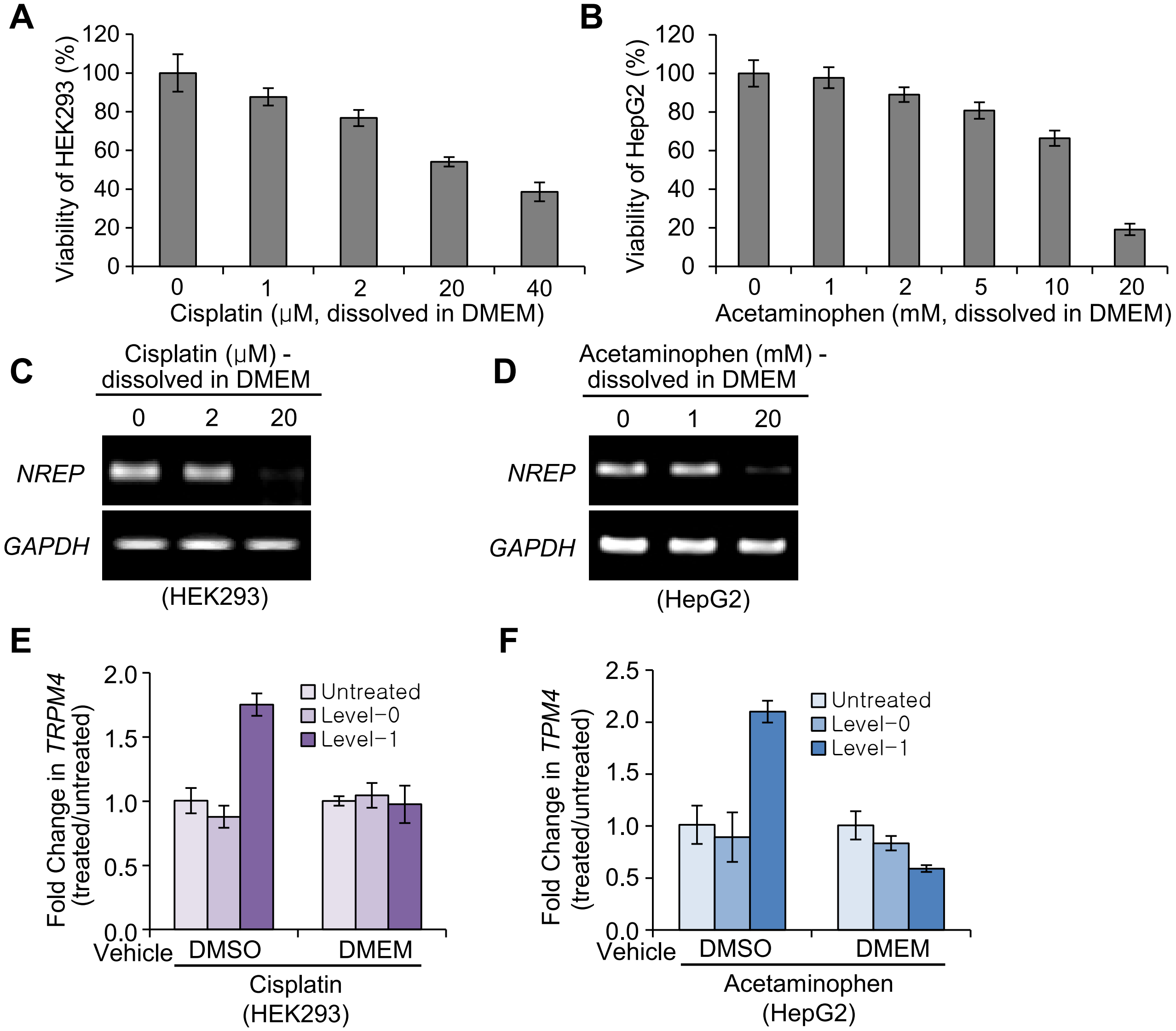

S4 Fig. Experimental validations identify NREP and CTSD as biomarkers of toxicity in human cell lines, related to Fig 5. (A, B) Dose-responsive viability of HEK293 (C) and HepG2 (D) cells exposed to cisplatin (A) or acetaminophen (B). Growth media (DMEM) was used to dissolve the compounds. Cell viability was measured by MTS assay. Error bars represent ± standard deviation of triplicate experiments. (C, D) *NREP* mRNA levels after exposure to the indicated concentrations of cisplatin for 72 h and acetaminophen for 48 h, determined by RT-PCR. (E, F) qRT-PCR assays for *TRPM4* (E) and *TPM4* (F). Y-axis indicates fold-changes in expression compared to chemically untreated samples (n = 5). Drug concentrations as in Fig 5G and H.
