# Supplementary material for: Meta-Analysis of Large-Scale Toxicogenomic Data Finds Neuronal Regeneration Related Protein and Cathepsin D to Be Novel Biomarkers of Drug-Induced Toxicity
Source: PLoS One. 2015 Sep 3;10(9):e0136698. doi: 10.1371/journal.pone.0136698 (PMC4559398; doi:10.1371/journal.pone.0136698)
Supplement: S1 Table — (PDF) [file pone.0136698.s005.pdf]

S1 Table. List of curated pathology terms for each organ according to the standardized ToxRefDB vocabulary

Pathology code	Kidney	Liver	Heart	Sum
Infiltration cellular	109	4,395	197	4,701
Necrosis	204	3,221	174	3,599
Glycogen depletion	0	3,085	0	3,085
Fatty change	0	2,328	91	2,419
Hypertrophy	1	2,195	91	2,287
Degeneration	32	1,960	206	2,198
Fibrosis	50	1,709	188	1,947
Mineralization	227	1,589	78	1,894
Hyperplasia	35	1,745	91	1,871
Mitosis	12	1,835	0	1,847
Eosinophilia	0	1,821	0	1,821
Dilatation	152	1,583	0	1,735
Clear cell focus	0	1,716	0	1,716
Hematopoiesis	0	1,703	0	1,703
Edema	0	1,595	91	1,686
Autolysis	82	1,601	0	1,683
Erythrophagocytosis	0	1,656	0	1,656
Atrophy	0	1,598	0	1,598
Lymphoma malignant	0	1,598	0	1,598
Multinucleated	0	1,598	0	1,598
Thrombus	0	1,598	0	1,598
Regeneration	228	42	0	270
Vacuolization	94	5	114	213
Basophilia	133	6	0	139
Inflammation	101	0	24	125
Bun	120	0	0	120
Hemorrhage	0	107	0	107
Cast	102	0	0	102
Cyst	98	0	0	98
Nephropathy	94	0	0	94
Congestion	0	92	0	92
Cytoplasmic condensation	0	92	0	92
Amyloid deposition	82	9	0	91
Hyaline droplet	82	0	0	82
Eosinophilic focus	10	17	0	27
Apoptosis	0	24	0	24
Proliferation	0	21	0	21
Cytoplasmic alteration	0	20	0	20
Nuclear alteration	0	17	0	17
Vacuolization cytoplasmic	0	11	0	11
Cardiomyopathy	0	0	10	10
Extramedullary hematopoiesis	0	6	0	6
Foci	0	6	0	6
Infarct	5	0	0	5
Karyomegaly	3	0	0	3
Accumulation, foam cell	0	2	0	2
Hepatodiaphragmatic nodule	0	1	0	1
