# Supplementary material for: Meta-Analysis of Large-Scale Toxicogenomic Data Finds Neuronal Regeneration Related Protein and Cathepsin D to Be Novel Biomarkers of Drug-Induced Toxicity
Source: PLoS One. 2015 Sep 3;10(9):e0136698. doi: 10.1371/journal.pone.0136698 (PMC4559398; doi:10.1371/journal.pone.0136698)
Supplement: S3 Table — (PDF) [file pone.0136698.s007.pdf]

S3 Table. Summary of the analyzed meta-data

**Organ**	**Training dataset**				**Test dataset**			
	Number of samples			Number of studies	Number of samples			Number of studies
	Untreated	Level-0	Level-1		Untreated	Level-0	Level-1	
Kidney	191	237	160	22	99	255	198	29
Liver	429	2,100	929	33	209	258	888	55
Heart	59	242	26	20	41	181	65	26
Data set	4,373			52	2,194			72

Untreated, untreated samples (pathology score < 0.5); level-0, innocuous treatment; level-1, toxic treatment.
