# Supplementary material for: Meta-Analysis of Large-Scale Toxicogenomic Data Finds Neuronal Regeneration Related Protein and Cathepsin D to Be Novel Biomarkers of Drug-Induced Toxicity
Source: PLoS One. 2015 Sep 3;10(9):e0136698. doi: 10.1371/journal.pone.0136698 (PMC4559398; doi:10.1371/journal.pone.0136698)
Supplement: S4 Table — (PDF) [file pone.0136698.s008.pdf]

S4 Table. The 44 previously known biomarkers

Gene Symbol
Spp1
Fhl1
Atp5i
Slc4a3
Adra2a
S100g
Tcf4
Kcnj12
Psma2
A2m
Anxa5
Ccng1
Cd24
Cd44
Clu
Cp
Ctss
Egf
Fn1
G6pc
Havcr1
Lcn2
Mgp
Klk1
Rgn
Tff3
Tnfrsf12a
Vim
Acaa1a
Alas2
Ccl2
Ctsl1
Cyp2c11
Fabp7
Gckr
Hmox1
Nfe2l2
Ntrk1
Ripk3
Slc7a1
Add1
Sult1c3
Phlda1
Vkorc1
