# Supplementary material for: Meta-Analysis of Large-Scale Toxicogenomic Data Finds Neuronal Regeneration Related Protein and Cathepsin D to Be Novel Biomarkers of Drug-Induced Toxicity
Source: PLoS One. 2015 Sep 3;10(9):e0136698. doi: 10.1371/journal.pone.0136698 (PMC4559398; doi:10.1371/journal.pone.0136698)
Supplement: S7 Table — (PDF) [file pone.0136698.s011.pdf]

S7 Table. List of 40 genes selected from sPLS-DA

**Meta-analysis comparison**	Gene Symbol
Untreated *vs*. treated (MA1)	Nrep
	Kifc1
	Ephx1
	Atrn
	Tbxa2r
	Pbld
	Col5a3
Organ *vs*. organ comparison (MA2)	Azgp1
	Nrg1
	Hao2
Level-0 *vs*. level-1 kidney (MA3)	Atp1b2
	Ralb
	Spp1
	Psmb10
	Aldh18a1
	Ctss
	Amigo2
	Tubb5
	Trpm4
	Cd86
	Sh3bgrl3
	Clu
	Ptprc
	Vim
	Il1rl2
	Psme2
	Lcp2
	Hmgb2
	Mepce
	Gja5
	Unc93b1
	Aifm3
	Slc15a3
	Lcp1
Level-0 *vs*. level-1 liver (MA4)	Ctsd
	Tpm4
	Rpl35a
Level-0 *vs*. level-1 heart (MA5)	Gpam
	Rxrg
	Pcp4l1
