# Supplementary material for: Meta-Analysis of Large-Scale Toxicogenomic Data Finds Neuronal Regeneration Related Protein and Cathepsin D to Be Novel Biomarkers of Drug-Induced Toxicity
Source: PLoS One. 2015 Sep 3;10(9):e0136698. doi: 10.1371/journal.pone.0136698 (PMC4559398; doi:10.1371/journal.pone.0136698)
Supplement: S8 Table — (PDF) [file pone.0136698.s012.pdf]

S8 Table. Summary statistics for the 18 toxicity biomarker candidates

**Comparison**	Gene	***p*-value**	**ES**	**Abs (ES)**
Untreated *vs*. treated (MA1)	Nrep	8.49×10^{-11}	-1.04	1.04
	Atrn	8.78×10^{-5}	-0.43	0.43
	Tbxa2r	5.14×10^{-5}	-0.27	0.27
	Kifc1	3.16×10^{-5}	-0.24	0.24
	Ephx1	1.46×10^{-4}	0.21	0.21
Level-0 *vs*. level-1 kidney (MA3)	Spp1	2.55×10^{-4}	1.22	1.22
	Ctss	5.45×10^{-6}	1.01	1.01
	Tubb5	1.48×10^{-4}	0.61	0.61
	Trpm4	5.99×10^{-5}	0.55	0.55
	Il1rl2	4.24×10^{-4}	-0.53	0.53
	Amigo2	4.06×10^{-5}	0.43	0.43
	Atp1b2	2.34×10^{-5}	-0.14	0.14
Level-0 *vs*. level-1 liver (MA4)	Ctsd	2.99×10^{-9}	0.9	0.9
	Tpm4	3.11×10^{-9}	0.83	0.83
	Rpl35a	9.05×10^{-6}	0.45	0.45
Level-0 *vs*. level-1 heart (MA5)	Gpam	3.44×10^{-5}	-1.06	1.06
	Rxrg	6.69×10^{-5}	-0.94	0.94
	Pcp4l1	2.39×10^{-4}	-0.88	0.88

p-value from meta-analysis, **ES:** effect size, **Abs:** absolute value
